# The isolation and identification of pathogenic fungi from *Tessaratoma papillosa* Drury (Hemiptera: Tessaratomidae)

**DOI:** 10.7717/peerj.3888

**Published:** 2017-10-06

**Authors:** Xiang Meng, Junjie Hu, Gecheng Ouyang

**Affiliations:** 1 Guangdong Key Laboratory of Animal Conservation and Resource Utilization, Guangdong Public Laboratory of Wild Animal Conservation and Utilization, Guangdong Institute of Applied Biological Resources, Guangzhou, Guangdong, China; 2 College of Life Science, Guangzhou University, Guangzhou, Guangdong, China

**Keywords:** *Tessaratoma papillosa*, Entomopathogenic fungi, Biological control, Pathogenicity

## Abstract

**Background:**

Litchi stink-bug, Tessaratoma papillosa Drury (Hemiptera: Tessaratomidae), is one of the most widespread and destructive pest species on *Litchi chinensis* Sonn and *Dimocarpus longan* Lour in Southern China. Inappropriate use of chemical pesticides has resulted in serious environmental problems and food pollution. Generating an improved Integrated Pest Management (IPM) strategy for litchi stink-bug in orchard farming requires development of an effective biological control agent. Entomopathogenic fungi are regarded as a vital ecological factor in the suppression of pest populations under field conditions. With few effective fungi and pathogenic strains available to control litchi stink-bug, exploration of natural resources for promising entomopathogenic fungi is warranted.

**Methods & Results:**

In this study, two pathogenic fungi were isolated from cadavers of adult *T. papillosa*. They were identified as *Paecilomyces lilacinus* and *Beauveria bassiana* by morphological identification and rDNA-ITS homogeneous analysis. Infection of *T. papillosa* with *B. bassiana* and *P. lilacinus* occurred initially from the antennae, metameres, and inter-segmental membranes. Biological tests showed that the two entomopathogenic fungi induced high mortality in 2^nd^ and 5^th^ instar nymphs of *T. papillosa*. *B. bassiana* was highly virulent on 2^nd^ instar nymphs of *T. papillosa*, with values for cadaver rate, LC_50_ and LT_50_ of 88.89%, 1.92 × 10^7^ conidia/mL and 4.34 days respectively.

**Discussion:**

This study provides two valuable entomopathogenic fungi from *T. papillosa*. This finding suggests that the highly virulent *P. lilacinus* and *B. bassiana* play an important role in the biocontrol of *T. papillosa* in China. These pathogenic fungi had no pollution or residue risk, and could provide an alternative option for IPM of litchi stink-bug.

## Introduction

Litchi stink-bug, *Tessaratoma papillosa* Drury (Hemiptera: Tessaratomidae), is a major pest on *Litchi chinensis* Sonn and *Dimocarpus longan* Lour in South East Asia and Southern China. The nymphs and adults feed on the tender branchlets, spica, and fruit for almost a year, reducing plant growth and causing flower and fruit drop, and can lead to wilting and even plant death (Waite & Hwang, 2002). Not only do the insects attack graminaceous plants, they also spread viruses between crops indirectly. These viruses, such as those causing witches’ broom diseases (Chen, Chen & Xu, 2001), can seriously affect the production of litchi and longan. When threatened, the bug releases large quantities of highly irritating and corrosive effluvial fluid from its scent gland (Zhao et al., 2012), which can burn or darken flowers, wilt young leaves, and even lead to brown spots and downy mildew on old leaves and fruits. These changes eventually reduce the edible and economical value of the fruit (Boontam & Leksawasdi, 1994). Chemical pesticides have been used to control *T. papillosa*, but pest control is poor and problems with pesticide residues on fruit and pest resistance to the chemicals used can seriously impact human health (Xu, 2005). Recently, an outbreak of litchi stink-bug occurred on litchi in Southern China during the 2013 to 2016 growing seasons. For these reasons, development of a non-hazard means of controlling *T. papillosa* on litchi has become urgent.

Biological control is one of the most widely used techniques for environmentally benign pest control (Ren & Chen, 2012). Many studies have focused on utilizing natural resources for pest control. Field research has identified many predatory enemies of *T. papillosa*, including *Tenodera sinensis* Saussure, *Hierodula patellifera* Serville, *Gampsocleis* sp., spiders, the South China tree-toad, and various birds (Li et al., 2013; Wang et al., 2015). The egg parasitoids *Anastatus japonicus* Ashmead and *Ooencyrtus corbetti* Ferr have been used as parasites in the biological control of *T. papillosa*, with some success (Zou, 2008; Li et al., 2013; Liu et al., 1995; Han et al., 1999; Chi, Xu & Wang, 2006). In addition, a method involving azadirachtin injection was also tested against *T. papillosa* and other pests of litchi (Marie, Konrad & Joachim, 2006). However, there are not enough natural enemies and plant extracts to control *T. papillosa* in the natural environment, and the large-scale production and application of natural enemies and plant extracts are usually restricted by artificial production conditions (Ren & Chen, 2012).

Entomopathogenic fungi, as alternatives to chemical control or as part of Integrated Pest Management (IPM) programs, are key factors in controlling pests. These fungi enter the insect by cuticular infection, and even produce toxins to aid invasion. They can infect different developmental stages of various kinds of insects, especially those with sucking mouthparts pests, as well as forest and soil pests (Wang et al., 2010). Many microbial insecticides are available for the biological control of insect pests, including *Metarhizium anisopliae*, *Beauveria bassiana*, *Paecilomycesfum osoroseus*, and *Verticillium lecanii* (Ferron, 1978; Ramakers & Samson, 1984; Fransen & Van Lanteren, 1993; Pingel & Lewis, 1996; Wang, 2012). However, few have been used successfully against *T. papillosa*. A strain of *Metarhizium anisopliae* was found to be highly pathogenic on *T. papillosa* when used at a rate of 1.0  ×10^7^ conidia/mL, with good insecticidal activity in the field (Jia, 2005). Fan et al. (2011) isolated four entomopathogenic fungi from soil samples of litchi orchards in South China, and two of them, *Metarhizium anisopliae* Bb07 and *Beauveria bassiana* Ma03, demonstrated good control of *T. papillosa* in field trials. The advantages of entomopathogenic fungi include a broad spectrum of diffusion effect, less likelihood of the pest developing resistance, ease of production, and greater specificity against target species (Ren & Chen, 2012). These considerations make it worthwhile to explore the natural availability of entomopathogenic fungus for use in the biological control of *T. papillosa*.

In this study, we investigated two pathogenic fungi from naturally infected *T. papillosa*, exhibiting signs of muscardine, in a litchi orchard. The aims of the study were (1) to collect and isolate new entomopathogenic fungi from *T. papillosa*, (2) to determine their pathogenicity on *T. papillosa*, and (3) to develop a biocontrol option for *T. papillosa*. It is hoped that this work will provide the basis for more detailed research on fungus-based pesticide products and their large-scale application for biological control of important pests in agriculture and forest systems.

## Materials and Methods

### Test strain

**Figure 1:**
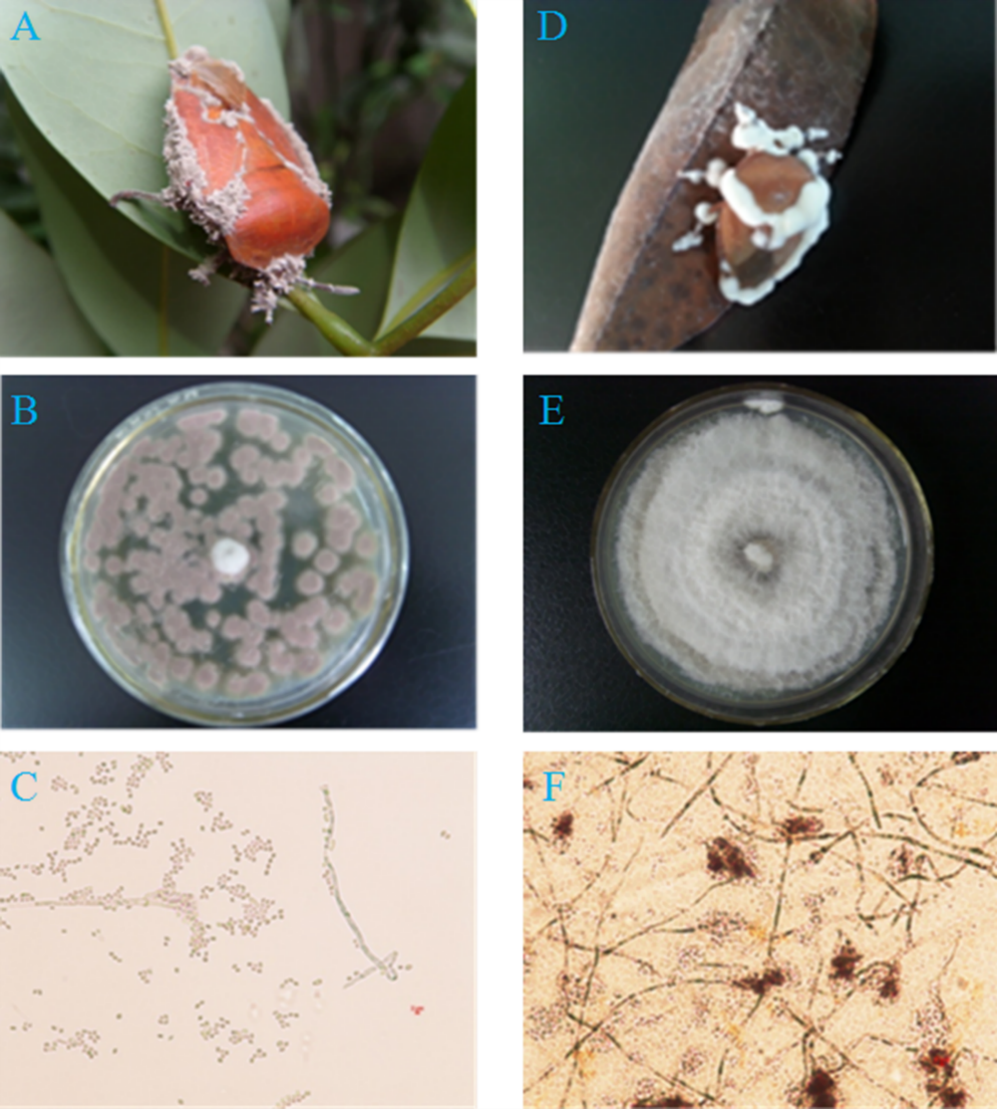
Two pathogenic fungi from cadavers of adult *T. papillosa* in a litchi orchard. (A, B, C) Fungal isolate Ta-01; (D, E, F) Fungal isolate Ta-02. (A, D) Adult *T. papillosa* invaded by *Paecilomyces lilacinus* and *Beauveria bassiana*, respectively; (B and E) Colonies of *P. lilacinus* and *B. bassiana* on PDA; (C and F) Conidioma shape and conidia of *P. lilacinus* and *B. bassiana*.

The fresh entomogenous fungi were collected from naturally infected adult *T. papillosa* exhibiting symptoms of muscardine on litchi leaves and branches from the litchi orchard (23.57N, 113.55E), which was located in Huangwei village, Conghua district, Guangzhou, China. Digital photomicrographs of the samples were taken (Fig. 1). Fungal strains were isolated from the adult *T. papillosa*, and cultured on potato dextrose agar (PDA) at 26 ± 1 °C and 60% RH in a biochemical incubator. Fungal strains exhibiting good growth and spore production traits were selected and purified for use as test strains. Samples of the strains were deposited in the laboratory of the Guangdong Institute of Applied Biological Resources, and the remainder of the strain was used for pathogenicity tests. The infected adults of *T. papillosa* were observed and recorded by microscope, and morphological identification based on phenotypic properties and morphology of the pathogenic fungus (Pu & Li, 1996; Liang et al., 2005; Luangsa-ard et al., 2005).

### Sequencing and phylogenetic analysis of fungal strain by ITS

Total DNA was isolated from each sample of the test strains using a fungal DNA kit and following the manufacturer’s instructions (E.Z.N.A. Fungal DNA Kit; Omega Bio-tek, Norcross, GA, USA). Purified DNA specimens were amplified with universal primers ITS1: 5′-TCCGTAGGTGAACCTGCGC-3′, ITS4: 5′-TCCTCCGCTTATTGATATGC-3′, and ITS5: 5′-GGAAGTAAAAGTCGTAACAAGG-3′. Each PCR was carried out in 25 µL, containing 0.5 µL *Taq* DNA polymerase, 2 µL dNTPs, 2.5 µL 10 × buffer, 1 µL each primer, 1 µL total DNA, and 17 µL ddH_2_O (TaKaRa, Kusatsu, Shiga, Japan). Amplification was performed in an Eppendorf Mastercycler ep (Eppendorf, Hamburg, Germany). ddH_2_O instead of template DNA was included as a negative control in each PCR to check for contamination. The reaction was performed using the following reaction cycles: initial denaturation at 94 °C for 5 min, followed by 35 cycles of denaturation at 94 °C for 30 s, annealing at 50 °C for 30 s, and extension at 72 °C for 1 min; then a final extension at 72 °C for 5 min. PCR products were visualized by 1.0% agarose gel electrophoresis, stained with GoldView in 0.5× TBE buffer (Sangon, Shanghai, China), and photographed under UV light. These PCR products were purified using a DNA gel extraction kit (Omega-Biotek, Norcross, GA, USA) following the manufacturer’s recommendation, then cloned into the pMD18-T vector (Takara, Kusatsu, Shiga, Japan), and sent to Invitrogen (Guangzhou, China) for complete sequencing.

The resulting sequences were checked and aligned using BioEdit sequence alignment editor 7.0.0 (Isis Pharmaceuticals, Inc., Carlsbad, CA, USA). The similarity of sequences compared with homologous sequences deposited in GenBank was calculated using “BLAST” tools on the National Center for Biotechnology Information (NCBI) website. Their amino acid sequences were edited and aligned using ClustalW (Thompson, Gibson & Higgins, 2002) multiple-alignment software (http://www.ebi.ac.uk/clustalw/index.html). The phylogenetic tree was constructed using the Neighbor-joining (NJ) method of MEGA 4 software (Tamura et al., 2007). Node support was assessed using a bootstrap procedure of 1,000 replicates (Felsenstein, 1985; Saitou & Nei, 1987).

### Pathogenicity testing of fungal strains

#### Preliminary screening of test strain pathogenicity in *T. papillosa*

*T. papillosa* (nymphs and adults) were collected from the net-house of our field experiment in the litchi orchard. They were transferred to the lab and fed with litchi leaves in a climatic chamber with 26 ± 1 °C, 60–80% RH, and a photoperiod of 14:10 h (L:D). Nymphs (2^nd^ and 5^th^ instars) and adults of *T. papillosa* were selected for uniformity in size before use in bioassays.

The two purified strains were cultivated on PDA medium in Petri dishes in a biochemical incubator (26 ± 1 °C , 60% RH) for five days. Conidia were harvested from plates, suspended in 0.05% Tween 80 (v/v), and shaken on a vortex mixer for 10 min. The conidial suspensions were then filtered, counted, and adjusted to the appropriate concentration for pathogenicity tests.

In the pre-selection bioassays, the two purified fungal strains were screened on *T. papillosa* to evaluate their pathogenicity. For both strains, a conidial suspension at a concentration of 1 × 10^8^ conidia/mL was appropriate for pathogenicity tests on *T. papillosa* within 15 days. The three growth stages of *T. papillosa* were soaked and infected with a conidial suspension of each test strain for 10 s. The insects were then removed, dried, and placed in sterilized Petri dishes (17 cm diameter and 3 cm high), with 10 insects in each Petri dish. Water on soaked cotton and litchi leaves was provided for the insects, after which they were placed in a climatic chamber. Negative controls consisted of the same densities of healthy *T. papillosa*, which were not treated with the fungal test strains. Each assay was replicated three times.

Infection of *T. papillosa* by the test strains was observed daily, and dead insects were removed and incubated to promote fungal growth. Mycelium and spores growing from the dead *T. papillosa* were examined to ensure they were the test strains used to inoculate the insects (Hu et al., 2007). The average mortalities of *T. papillosa* caused by the two tested strains were calculated, after which the corrected mortality was calculated. Statistical significance was tested using one-way ANOVA with a post hoc LSD test (SPSS17.0). Results were considered statistically significant when *p*-values were <0.05.

#### Pathogenicity determination of the test strains

The two highly virulent strains from preliminary screening tests were used to determine their LC_50_ values on 2^nd^ and 5^th^ instar nymphs, and adult *T. papillosa*. Conidial suspensions of the test strains were diluted with sterile distilled water to give five concentrations (1 × 10^8^, 5 × 10^7^, 2.5 × 10^7^, 1.25 × 10^7^, and 6.25 × 10^6^ conidia/mL); sterile water was used as a blank control. Ten insects were treated with each concentration of the conidial suspension, and each assay was replicated three times. Corrected mortalities were calculated, the LC_50_ and LT_50_ were determined using a biological assay procedure of generalized linear regreesion method with binomial errors and logit link function in statistical software DPS9.50 (Tang & Zhang, 2012).

## Results

### Morphological identification of infected *T. papillosa*

We separated and purified two strains (Ta-01, Ta-02) from adult *T. papillosa* in the litchi orchard (Fig. 1). The artificial infection test using adult *T. papillosa* showed that both strains can cause infection, after which the insects moved slowly and suffered a slight spasm after 48–72 h. Strain Ta-01 caused infected *T. papillosa* to feed less actively, and they clung tightly to litchi branches until they became stiff and died. Microscopic observation showed that gray mycelium grew in metamere and inter-segmental membranes of dead *T. papillosa* after seven days or longer. Insects infected with strain Ta-02 died and exhibited white hyphae growing from their antennae and inter-segmental membranes after they had been cultured for seven days. Morphological identification indicated that strain Ta-01 strain is *Paecilomyces lilacinus* and strain Ta-02 is *Beauveria bassiana* (Junior et al., 1997; Fang, Zhang & Liu, 2001; Li, Liu & Huang, 2004; Lu et al., 2007).

### Sequencing of ITS and phylogenetic analysis

**Figure 2:**
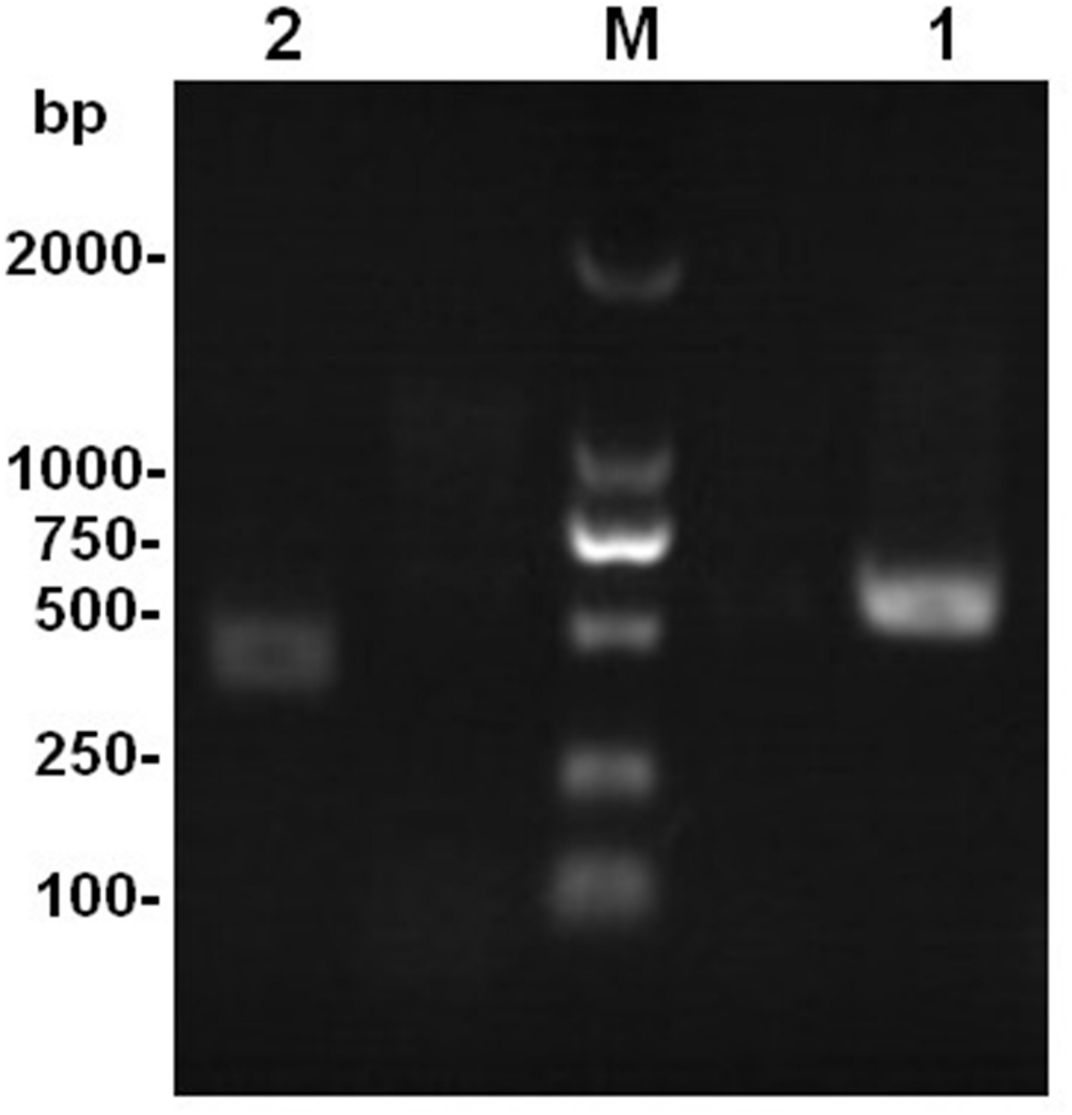
The amplification product of the rDNA-ITS gene from strains Ta-01 and Ta-02. M, DNA marker; 1, Ta-01; 2, Ta-02.

**Figure 3:**
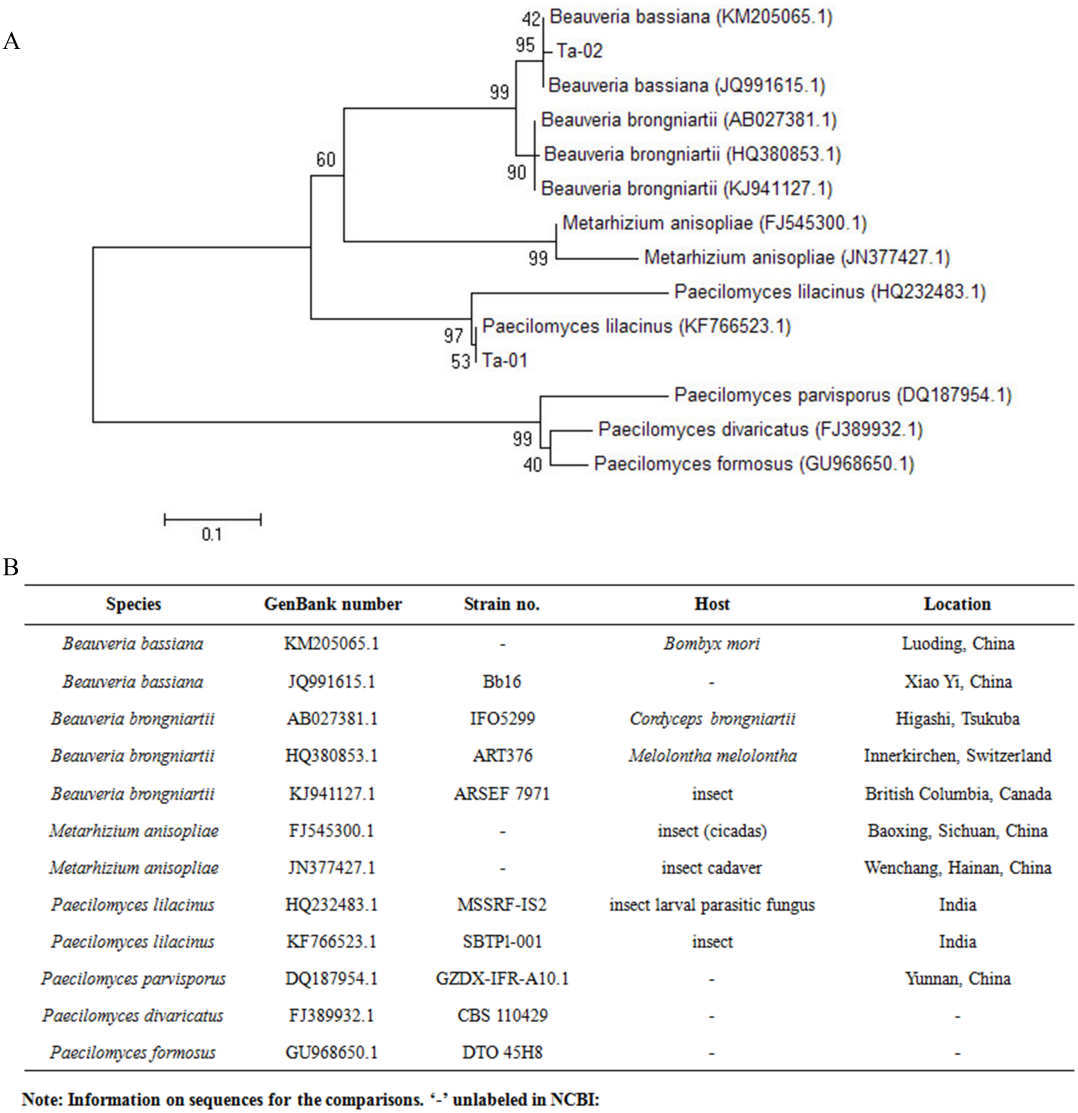
Phylogenetic tree based on the 28S rDNA sequence of strains Ta-01 and Ta-02. MEGA 4 was used to construct the phylogenetic tree. Bootstrap analyses from 1,000 replications are shown by each branch.

The results of rDNA-ITS sequencing of the two strains showed that 559 and 451 bp of special DNA fragments were sequenced (Fig. 2). The resulting sequences were compared with sequences of 18S rDNA accessed in GenBank using BLAST (Fig. 3). Phylogenetic analysis indicated that the obtained sequence for Ta-01 shares 99% homology with *Paecilomyces lilacinus* (KF766523.1), forming a cluster. The sequence for strain Ta-02 formed a cluster with *Beauveria bassiana* (JQ991615.1, KM205065.1), with a homology of 99%. Together, morphological identification and molecular identification showed that strains Ta-01 and Ta-02 are *P. lilacinus* and *B. bassiana*, respectively.

### Pathogenicity analysis of strains

In this study, *P. lilacinus* and *B. bassiana* were isolated from *T. papillosa* and their pathogenicities were determined. The bioassay results showed that both strains were pathogenic towards *T. papillosa*, with *B. bassiana* more virulent than *P. lilacinus*, with a corrected mortality rate of infected 2^nd^ instar nymphs, 5^th^ instar nymphs, and adult *T. papillosa* within 10 days of 88.89 ± 6.42%, 75.56 ± 4.44%, and 65.56 ± 2.94%, respectively; there was a significant difference in mortality rate between infected 2^nd^ instar nymphs and adult *T. papillosa* caused by infection with *B. bassiana* (*F* = 3.364, *df* = 5, 18, *P* < 0.05). The corrected mortality rates of infected 2^nd^ instar nymphs, 5^th^ instar nymphs, and adult *T. papillosa* caused by infection with *P. lilacinus* were 78.15 ± 6.74%, 68.89 ± 5.88%, and 63.33 ± 3.33%, which were not significantly different (*F* = 3.364, *df* = 5, 18, *P* > 0.05) (Table 1). LC_50_ values for *B. bassiana* (Table 2) acting on *T. papillosa* 2^nd^ and 5^th^ instar nymphs after 10 days were 1.43 × 10^7^ conidia/mL and 1.36 × 10^7^ conidia/mL, respectively, whereas LC_50_ values for *T. papillosa* infected by *P. lilacinus* 2^nd^ instar nymphs, 5^th^ instar nymphs, and adults were 1.92 × 10^7^ conidia/mL, 1.95 × 10^7^ conidia/mL, and 2.54 × 10^7^ conidia/mL, respectively (Table 2).

LT_50_ values for the two test strains are shown in Table 3. The results show that the LT_50_ for *B. bassiana* on 2^nd^ instar nymphs of *T. papillosa* was 4.34 days, whichwas significantly lower than the LT_50_ values for *P. lilacinus*. These results imply that the two entomopathogenic fungi were most effective in causing host death at the 2^nd^ instar nymph stage.

## Discussion

Research on biological approaches to controlling major pests of the litchi tree is a basic safeguard to the production of pollution-free litchi. Entomopathogenic fungi are regarded as vital ecological factors in suppressing pest populations in the field. They have broad-spectrum activity, good persistence in the soil and a unique way of infecting their insect hosts. In addition, they produce toxins, they are easy to mass-produce, and development of host resistance against entomopathogenic fungi is unlikely. Several studies have examined the ability of various treatments, including soil-dwelling fungi from insect cadavers and biological agents in combination with fungi, to control *T. papillosa* (Huang et al., 2009; Fan et al., 2011). In the present research, we discovered and identified two new strains of *P. lilacinus* and *B. bassiana*. Our results indicate that their use offers a promising strategy for the biological control of *T. papillosa* in the protected Litchi orchard ecosystem.

**Table 1:** Pathogenicity of *Paecilomyces lilacinus* and *Beauveria bassiana* against different developmental stages of *T. papillosa* 10 d after treatment (mean ± SE) at the concentration of 10^8^ conidia/mL.

Species	Insect stages	Sample (*N*)	Mortality (%)	Corrected mortality (%)
*Paecilomyces lilacinus*	2^nd^ instar nymph	10	80.00	78.15 ± 6.74 ab
	5^th^ instar nymph	10	70.00	68.89 ± 5.88 b
	Adult	10	63.33	63.33 ± 3.33 b
*Beauveria bassiana*	2^nd^ instar nymph	10	90.00	88.89 ± 6.42 a
	5^th^ instar nymph	10	76.67	75.56 ± 4.44 ab
	Adult	10	66.67	65.56 ± 2.94 b

**Notes.**

Different letters following the data within a column indicate a significant difference at *P* < 0.05.

**Table 2:** Pathogenicity regression equations for LC_50_ values of *Paecilomyces lilacinus and Beauveria bassiana* against different developmental stages of *T. papillosa* 10 d after treatment.

Species	Insect stages	Slope	Correlation coefficient	*P*	LC_50_ and 95% confidence interval (×10^7^ conidia/mL)
*Paecilomyces lilacinus*	2^nd^ instar nymph	1.2116	0.9825	0.0028	1.92 (1.54–2.38)
	5^th^ instar nymph	0.8712	0.9494	0.0135	1.95 (1.33–2.84)
	Adult	0.7801	0.9398	0.0176	2.54 (1.70–3.81)
*Beauveria bassiana*	2^nd^ instar nymph	1.3552	0.9679	0.0069	1.43 (1.03–2.00)
	5^th^ instar nymph	0.9437	0.9725	0.0054	1.36 (0.99–1.85)
	Adult	0.7394	0.9532	0.0121	2.07 (1.45–2.97)

**Table 3:** Pathogenicity regression equations for LT_50_ values of *Paecilomyces lilacinus and Beauveria bassiana* against different developmental stages of *T. papillosa* (1 × 10^8^ conidia/mL).

Species	Insect stages	Slope	Correlation coefficient	*P*	LT_50_ and 95% confidence interval (d)
*Paecilomyces lilacinus*	2^nd^ instar nymph	4.8383	0.9316	0.0069	6.00 (5.18–13.44)
	5^th^ instar nymph	2.4650	0.9950	0.0001	6.13 (5.55–6.77)
	Adult	4.2005	0.9223	0.0088	8.35 (5.18–13.44)
*Beauveria bassiana*	2^nd^ instar nymph	3.0735	0.9880	0.0002	4.34 (3.76–5.01)
	5^th^ instar nymph	4.8975	0.9503	0.0036	6.41 (4.61–8.915)
	Adult	4.4580	0.9432	0.0047	7.85 (5.32–11.57)

We isolated two pathogenic fungi from the cadavers of adult *T. papillosa* by conventional morphological identification methods. morphological identification showed that strain Ta-01 is *Paecilomyces lilacinus* and strain Ta-02 is *Beauveria bassiana*. Subsequent phylogenetic analysis using rDNA-ITS indicated that strain Ta-01 formed a cluster with *P. lilacinus* in the NCBI database, sharing 99% homology with *P. lilacinus* (KF766523.1). Strain Ta-02 displayed similar morphological characteristics in terms of color and size to *B. bassiana*, and showed a close relationship, supported by a 99% bootstrap value, with *B. bassiana* (JQ991615.1, KM205065.1). The results were consistent with the morphological identification. Our studies provide two new strains for the fungal control of *T. papillosa* and enrich the diversity of available resources of entomogenous fungi.

Fungal strains with a high virulence and good growth characteristics provide the basis for pest biocontrol. In general, the greater mortality rate and lower LC_50_ and LT_50_ values imply a higher degree of pathogenicity (Wekesa et al., 2005; Cheng et al., 2016). According to the results from the present study, we observed that the *T. apapillosa* infected with *B. bassiana* and *P. lilacinus* were initially and rapidly infected from its antenna, metamere and inter-segmental membranes. The results presented here show that both strains of *B. bassiana* and *P. lilacinus* are pathogenic towards *T. papillosa*, giving high mortality values, although the fungi differ significantly from one another. Mortality and virulence were affected by insect developmental stage, with *B. bassiana* showing the higher virulence of the two fungal strains. For the 2^nd^ instar nymph of *T. papillosa*, the mortality rate resulting from infection with *B. bassiana* reached 88.89 ± 6.42% at 10 days, which was significantly greater than that of *P. lilacinus* (78.15 ± 6.74%). *B. bassiana* LC_50_ and LT_50_ values of 1.43 × 10^7^ conidia/mL at 10 days and 4.34 days at 1 × 10^8^ conidia/mL, respectively, were lower than those of *P. lilacinus* (1.92 × 10^7^ conidia/mL for the LC_50_ and 6.00 days for the LT_50_). Fan et al. (2011) reported that *B. bassiana* strain Bb07, isolated from soil samples from litchi orchards in South China, gave an 89.66% mortality rate against 3^rd^ instar nymphs of *T. papillosa*. This is consistent with our results, although there are some differences; for example, in their work, the LC_50_ value at 14 days was 2.20 × 10^6^ conidia/mL^−1^ and the LT_50_ value after 6.1 days was 1 × 10^7^ conidia/mL^−1^. The effects of *P. lilacinus* on *T. papillosa* had been observed in 1959 (Lin, 1959). However, these earlier studies provided little detail to support the reported control of the pest on *T. papillosa* (Chen, Li & Liu, 1993). The new data provided in the present study supplement data provided by the previous studies. In addition, previous studies demonstrated that strains Ma01 and Ma03 of *M. anisopliae* were also active against *T. papillosa* with mortality rates of 73.13% and 93.33%, respectively. The strains identified in the present work show potential to control *T. papillosa*. They can be developed into commercial preparations for biocontrol of *T. papillosa* in litchi orchards, providing good conidial production can be achieved.

Entomopathogenic fungi can control insect pests and, as a result, have potential for use in biological control systems. They offer the advantages of no pollution, safety, and avoiding pesticide resistance, and they can sustainably control insects during their reproductive phase, thereby reducing pest populations and the risks of epidemics. However, in the long-term, the effectiveness of entomopathogenic fungi as biocontrol agents can decline as a result of strain degradation, variation, rejuvenation, and contamination (Vey et al., 1993; Huang & Li, 1994; Zimmenermann, 2007). The culture and storage of *B. bassiana* and *P. lilacinus*, correlating the virulence of *B. bassiana* and *P. lilacinus* with age of *T. papillosa*, ambient temperature and humidity, and other factors, and the field application and effectiveness of *B. bassiana* and *P. lilacinus* in controlling *T. papillosa* remain to be examined.

##  Supplemental Information

10.7717/peerj.3888/supp-1 Supplemental Information 1 LC_50_ and LT_50_

10.7717/peerj.3888/supp-2 Supplemental Information 2 Raw data

10.7717/peerj.3888/supp-3 Supplemental Information 3 The mortality results

10.7717/peerj.3888/supp-4 Supplemental Information 4 Sequence data

## Additional Information and Declarations

### Data Availability

The following information was supplied regarding data availability: The raw data has been supplied as a Supplementary File.
