# Supplementary material for: The isolation and identification of pathogenic fungi from Tessaratoma papillosa Drury (Hemiptera: Tessaratomidae)
Source: PeerJ. 2017 Oct 6;5:e3888. doi: 10.7717/peerj.3888 (PMC5633030; doi:10.7717/peerj.3888)
Supplement: Supplemental Information 2 [file peerj-05-3888-s002.docx]

**Bioactivity assay**

| Species | Insect stages | repetition | Sample(N) | Mortality(N) | Mortality (%) | Corrected mortality (%) |
| --- | --- | --- | --- | --- | --- | --- |
| *Beauveria bassiana* | young nymph | 1-1 | 10 | 10 | 100 | 100 |
|  |  | 1-2 | 10 | 8 | 80 | 77.77778 |
|  |  | 1-3 | 10 | 9 | 90 | 88.88889 |
|  |  | 1Ck-1 | 10 | 1 | 10 |  |
|  |  | 1Ck-2 | 10 | 1 | 10 |  |
|  |  | 1Ck-3 | 10 | 1 | 10 |  |
|  | old nymph | 2-1 | 10 | 8 | 80 | 80 |
|  |  | 2-2 | 10 | 7 | 70 | 66.66667 |
|  |  | 2-3 | 10 | 8 | 80 | 80 |
|  |  | 2Ck-1 | 10 | 0 | 0 |  |
|  |  | 2Ck-2 | 10 | 1 | 10 |  |
|  |  | 2Ck-3 | 10 | 0 | 0 |  |
|  | adult | 3-1 | 10 | 6 | 60 | 60 |
|  |  | 3-2 | 10 | 7 | 70 | 66.66667 |
|  |  | 3-3 | 10 | 7 | 70 | 70 |
|  |  | 3Ck-1 | 10 | 0 | 0 |  |
|  |  | 3Ck-2 | 10 | 1 | 10 |  |
|  |  | 3Ck-3 | 10 | 0 | 0 |  |
| *Paecilomyces lilacinus* | young nymph | 4-1 | 10 | 9 | 90 | 90 |
|  |  | 4-2 | 10 | 8 | 80 | 77.77777778 |
|  |  | 4-3 | 10 | 7 | 70 | 66.66666667 |
|  |  | 4Ck-1 | 10 | 0 | 0 |  |
|  |  | 4Ck-2 | 10 | 1 | 10 |  |
|  |  | 4Ck-3 | 10 | 1 | 10 |  |
|  | old nymph | 5-1 | 10 | 8 | 80 | 80 |
|  |  | 5-2 | 10 | 6 | 60 | 60 |
|  |  | 5-3 | 10 | 7 | 70 | 66.66666667 |
|  |  | 6Ck-1 | 10 | 0 | 0 |  |
|  |  | 6Ck-2 | 10 | 0 | 0 |  |
|  |  | 6Ck-3 | 10 | 1 | 10 |  |
|  | adult | 7-1 | 10 | 6 | 60 | 60 |
|  |  | 7-2 | 10 | 7 | 70 | 70 |
|  |  | 7-3 | 10 | 6 | 60 | 60 |
|  |  | 7Ck-1 | 10 | 0 | 0 |  |
|  |  | 7Ck-2 | 10 | 0 | 0 |  |
|  |  | 7Ck-3 | 10 | 0 | 0 |  |

ANOVA

| 2014-4-24 16:31:54  Result | | | | | | | |
| --- | --- | --- | --- | --- | --- | --- | --- |
| Treatment | sample size | average | SD | SE | 95%confidence interval | |  |
| 1 | 3 | 88.8889 | 11.1111 | 6.415 | 61.2874 | 116.4904 |  |
| 2 | 3 | 75.5556 | 7.698 | 4.4444 | 56.4327 | 94.6785 |  |
| 3 | 3 | 65.5556 | 5.0918 | 2.9397 | 52.9069 | 78.2042 |  |
| 4 | 3 | 78.1482 | 11.6711 | 6.7383 | 49.1556 | 107.1407 |  |
| 5 | 3 | 68.8889 | 10.1835 | 5.8794 | 43.5917 | 94.1861 |  |
| 6 | 3 | 63.3333 | 5.7735 | 3.3333 | 48.9912 | 77.6755 |  |
| table of variance analysis | | | | | | | |
| Source of variation | quadratic sum | df | mean square | F value | palue |  |  |
| among treatments | 1350.96 | 5 | 270.1921 | 3.364 | 0.0395 |  |  |
| within treatments | 963.7858 | 12 | 80.3155 |  |  |  |  |
| total variation | 2314.746 | 17 |  |  |  |  |  |
| Duncan |  |  |  |  |  |  |  |
| No. | average | 1 | 4 | 2 | 5 | 3 | 6 |
| 1 | 88.8889 |  | 0.1679 | 0.1079 | 0.0256 | 0.0127 | 0.0081 |
| 4 | 78.1482 | 10.7407 |  | 0.7293 | 0.2517 | 0.1362 | 0.0891 |
| 2 | 75.5556 | 13.3333 | 2.5926 |  | 0.3802 | 0.2177 | 0.1471 |
| 5 | 68.8889 | 20 | 9.2593 | 6.6667 |  | 0.6569 | 0.4842 |
| 3 | 65.5556 | 23.3333 | 12.5926 | 10 | 3.3333 |  | 0.7666 |
| 6 | 63.3333 | 25.5556 | 14.8148 | 12.2222 | 5.5556 | 2.2222 |  |
| Different letters fllowing the data within a column indicate significant difference | | | | | | | |
| Treatment | average | 5% level |  | 1% level |  |  |  |
| 1 | 88.8889 | a |  | A |  |  |  |
| 4 | 78.1482 | ab |  | AB |  |  |  |
| 2 | 75.5556 | ab |  | AB |  |  |  |
| 5 | 68.8889 | b |  | AB |  |  |  |
| 3 | 65.5556 | b |  | AB |  |  |  |
| 6 | 63.3333 | b |  | B |  |  |  |

**LC50 Values**

| **2013-7-8(10d) *Beauveria bassiana*** | | | | | | | |
| --- | --- | --- | --- | --- | --- | --- | --- |
| **Insect stages** | **Treatment** | **sample No** | **Mortality (N)** | | | **mortality (%)** | **Corrected mortality (%)** |
|  |  |  | **repetition1** | **repetition2** | **repetition3** |  |  |
| young nymph | 6.25 | **10** | **3** | **6** | **4** | **43.33333** | **37.03704** |
|  | 12.5 | **10** | **4** | **4** | **5** | **43.33333** | **37.03704** |
|  | 25 | **10** | **8** | **7** | **6** | **70** | **66.66667** |
|  | 50 | **10** | **8** | **9** | **6** | **76.66667** | **74.07407** |
|  | 100 | **10** | **10** | **8** | **9** | **90** | **88.88889** |
|  | **CK** | **10** | **1** | **1** | **1** | **10** | **0** |
| old nymph | 6.25 | **10** | **4** | **4** | **3** | **36.66667** | **34.48276** |
|  | 12.5 | **10** | **5** | **3** | **7** | **50** | **48.27586** |
|  | 25 | **10** | **5** | **7** | **8** | **66.66667** | **65.51724** |
|  | 50 | **10** | **6** | **9** | **7** | **73.33333** | **72.41379** |
|  | 100 | **10** | **8** | **7** | **8** | **76.66667** | **75.86207** |
|  | **CK** | **10** | **0** | **1** | **0** | **3.333333** | **0** |
| adult | 6.25 | **10** | **4** | **3** | **3** | **33.33333** | **31.03448** |
|  | 12.5 | **10** | **4** | **3** | **7** | **46.66667** | **44.82759** |
|  | 25 | **10** | **5** | **6** | **7** | **60** | **58.62069** |
|  | 50 | **10** | **5** | **7** | **7** | **63.33333** | **62.06897** |
|  | 100 | **10** | **6** | **7** | **7** | **66.66667** | **65.51724** |
|  | **CK** | **10** | **0** | **1** | **0** | **3.333333** | **0** |

| **Result of young nymph 2014-4-25 16:39:46** | | | | | | | |
| --- | --- | --- | --- | --- | --- | --- | --- |
|  | **Dose** | **logarithm of doses** | **treatment effect** | **probit value** |  |  |  |
|  | **6.25** | **0.7959** | **37.037** | **4.6691** |  |  |  |
|  | **12.5** | **1.0969** | **37.037** | **4.6691** |  |  |  |
|  | **25** | **1.3979** | **66.6667** | **5.4307** |  |  |  |
|  | **50** | **1.699** | **74.0741** | **5.6456** |  |  |  |
|  | **100** | **2** | **88.8889** | **6.2206** |  |  |  |
|  |  |  |  |  |  |  |  |
|  | **regression intercept A** | **SE** | **regression coefficientB** | **SE** | **correlation coefficient** | **F- inspection value** | **p-** **value** |
|  | **3.4326** | **0.2969** | **1.3552** | **0.2032** | **0.9679** | **44.4929** | **0.0069** |
| **LD50** | **Logarithmic concentration =** | **1.1566** | **95%** **confidence interval** | **1.0128** | **~** | **1.3004** |  |
|  | **concentration =** | **14.3419** | **95%** **confidence interval** | **10.2994** | **~** | **19.9712** |  |
| **Result of** **old nymph 2014-4-25 16:41:20** | | | | | | | |
|  | **Dose** | **logarithm of doses** | **treatment effect** | **probit value** |  |  |  |
|  | **6.25** | **0.7959** | **34.4828** | **4.6007** |  |  |  |
|  | **12.5** | **1.0969** | **48.2759** | **4.9568** |  |  |  |
|  | **25** | **1.3979** | **65.5172** | **5.3993** |  |  |  |
|  | **50** | **1.699** | **72.4138** | **5.5952** |  |  |  |
|  | **100** | **2** | **75.8621** | **5.7019** |  |  |  |
|  |  |  |  |  |  |  |  |
|  | **regression intercept A** | **SE** | **regression coefficientB** | **SE** | **correlation coefficient** | **F- inspection value** | **p-** **value** |
|  | **3.9315** | **0.1905** | **0.9437** | **0.1304** | **0.9725** | **52.3793** | **0.0054** |
| **LD50** | **Logarithmic concentration =** | **1.1322** | **95%** **confidence interval** | **0.9963** | **~** | **1.2681** |  |
|  | **concentration =** | **13.5586** | **95%** **confidence interval** | **9.9153** | **~** | **18.5405** |  |
| **Result of** **adult 2014-4-25 16:42:00** | | | | | | | |
|  | **Dose** | **logarithm of doses** | **treatment effect** | **probit value** |  |  |  |
|  | **6.25** | **0.7959** | **31.0345** | **4.5051** |  |  |  |
|  | **12.5** | **1.0969** | **44.8276** | **4.87** |  |  |  |
|  | **25** | **1.3979** | **58.6207** | **5.2178** |  |  |  |
|  | **50** | **1.699** | **62.069** | **5.3073** |  |  |  |
|  | **100** | **2** | **65.5172** | **5.3993** |  |  |  |
|  |  |  |  |  |  |  |  |
|  | **regression intercept A** | **SE** | **regression coefficientB** | **SE** | **correlation coefficient** | **F- inspection value** | **p-** **value** |
|  | **4.0263** | **0.1978** | **0.7394** | **0.1354** | **0.9532** | **29.8361** | **0.0121** |
| **LD50** | **Logarithmic concentration =** | **1.3169** | **95%** **confidence interval** | **1.1614** | **~** | **1.4724** |  |
|  | **concentration =** | **20.7452** | **95%** **confidence interval** | **14.5016** | **~** | **29.677** |  |

| **2013-7-8(10d) *Beauveria bassiana*** | | | | | | | |
| --- | --- | --- | --- | --- | --- | --- | --- |
| **Insect stages** | **Treatment** | **sample No** | **Mortality (N)** | | | **mortality (%)** | **Corrected mortality (%)** |
|  |  |  | **repetition1** | **repetition2** | **repetition3** |  |  |
| young nymph | 6.25 | **10** | **3** | **6** | **4** | **43.33333** | **37.03704** |
|  | 12.5 | **10** | **4** | **4** | **5** | **43.33333** | **37.03704** |
|  | 25 | **10** | **8** | **7** | **6** | **70** | **66.66667** |
|  | 50 | **10** | **8** | **9** | **6** | **76.66667** | **74.07407** |
|  | 100 | **10** | **10** | **8** | **9** | **90** | **88.88889** |
|  | **CK** | **10** | **1** | **1** | **1** | **10** | **0** |
| old nymph | 6.25 | **10** | **4** | **4** | **3** | **36.66667** | **34.48276** |
|  | 12.5 | **10** | **5** | **3** | **7** | **50** | **48.27586** |
|  | 25 | **10** | **5** | **7** | **8** | **66.66667** | **65.51724** |
|  | 50 | **10** | **6** | **9** | **7** | **73.33333** | **72.41379** |
|  | 100 | **10** | **8** | **7** | **8** | **76.66667** | **75.86207** |
|  | **CK** | **10** | **0** | **1** | **0** | **3.333333** | **0** |
| adult | 6.25 | **10** | **4** | **3** | **3** | **33.33333** | **31.03448** |
|  | 12.5 | **10** | **4** | **3** | **7** | **46.66667** | **44.82759** |
|  | 25 | **10** | **5** | **6** | **7** | **60** | **58.62069** |
|  | 50 | **10** | **5** | **7** | **7** | **63.33333** | **62.06897** |
|  | 100 | **10** | **6** | **7** | **7** | **66.66667** | **65.51724** |
|  | **CK** | **10** | **0** | **1** | **0** | **3.333333** | **0** |

| **Result of young nymph 2014-4-25 16:43:13** | | | | | | | | | | | |
| --- | --- | --- | --- | --- | --- | --- | --- | --- | --- | --- | --- |
|  | **Dose** | **logarithm of doses** | **treatment effect** | | **probit value** | |  | |  | |  |
|  | **6.25** | **0.7959** | **28.5714** | | **4.4341** | |  | |  | |  |
|  | **12.5** | **1.0969** | **35.7143** | | **4.6339** | |  | |  | |  |
|  | **25** | **1.3979** | **60.7143** | | **5.2719** | |  | |  | |  |
|  | **50** | **1.699** | **71.4286** | | **5.5659** | |  | |  | |  |
|  | **100** | **2** | **78.5714** | | **5.7916** | |  | |  | |  |
|  |  |  |  | |  | |  | |  | |  |
|  | **regression intercept A** | **SE** | **regression coefficientB** | | **SE** | | **correlation coefficient** | | **F- inspection value** | | **p-** **value** |
|  | **3.4458** | **0.1936** | **1.2116** | | **0.1325** | | **0.9825** | | **83.6009** | | **0.0028** |
| **LD50** | **Logarithmic concentration =** | **1.2828** | **95%** **confidence interval** | | **1.1883** | | **~** | | **1.3774** | |  |
|  | **concentration =** | **19.1786** | **95%** **confidence interval** | | **15.4269** | | **~** | | **23.8426** | |  |
| **Result of old nymph 2014-4-25 16:43:46** | | | | | | | | | | | |
|  | **Dose** | **logarithm of doses** | **treatment effect** | **probit value** | |  | |  | |  | |
|  | **6.25** | **0.7959** | **27.5862** | **4.4048** | |  | |  | |  | |
|  | **12.5** | **1.0969** | **48.2759** | **4.9568** | |  | |  | |  | |
|  | **25** | **1.3979** | **58.6207** | **5.2178** | |  | |  | |  | |
|  | **50** | **1.699** | **65.5172** | **5.3993** | |  | |  | |  | |
|  | **100** | **2** | **68.9655** | **5.4949** | |  | |  | |  | |
|  |  |  |  |  | |  | |  | |  | |
|  | **regression intercept A** | **SE** | **regression coefficientB** | **SE** | | **correlation coefficient** | | **F- inspection value** | | **p-** **value** | |
|  | **3.8768** | **0.2431** | **0.8712** | **0.1663** | | **0.9494** | | **27.4361** | | **0.0135** | |
| **LD50** | **Logarithmic concentration =** | **1.2892** | **95%** **confidence interval** | **1.1248** | | **~** | | **1.4536** | |  | |
|  | **concentration =** | **19.4636** | **95%** **confidence interval** | **13.3294** | | **~** | | **28.4208** | |  | |
| **Result of** **adult 2014-4-25 16:44:12** | | | | | | | | | | | |
|  | **Dose** | **logarithm of doses** | **treatment effect** | **probit value** | |  | |  | |  | |
|  | **6.25** | **0.7959** | **26.6667** | **4.3771** | |  | |  | |  | |
|  | **12.5** | **1.0969** | **43.3333** | **4.8321** | |  | |  | |  | |
|  | **25** | **1.3979** | **56.6667** | **5.1679** | |  | |  | |  | |
|  | **50** | **1.699** | **60** | **5.2533** | |  | |  | |  | |
|  | **100** | **2** | **63.3333** | **5.3407** | |  | |  | |  | |
|  |  |  |  |  | |  | |  | |  | |
|  | **regression intercept A** | **SE** | **regression coefficientB** | **SE** | | **correlation coefficient** | | **F- inspection value** | | **p-** **value** | |
|  | **3.9036** | **0.2394** | **0.7801** | **0.1638** | | **0.9398** | | **22.6761** | | **0.0176** | |
| **LD50** | **Logarithmic concentration =** | **1.4053** | **95%** **confidence interval** | **1.2301** | | **~** | | **1.5806** | |  | |
|  | **concentration =** | **25.4299** | **95%** **confidence interval** | **16.9861** | | **~** | | **38.0712** | |  | |
