# Supplementary material for: The isolation and identification of pathogenic fungi from Tessaratoma papillosa Drury (Hemiptera: Tessaratomidae)
Source: PeerJ. 2017 Oct 6;5:e3888. doi: 10.7717/peerj.3888 (PMC5633030; doi:10.7717/peerj.3888)
Supplement: Supplemental Information 3 [file peerj-05-3888-s003.pdf]

the mortality results by two method (SPSS,DPS)

Corrected mortality

			mortality(from raw data)		
			repetition1	2	3
P. lilacinu	2nd instar nymj	1	90	77.78	66.67
	5th instar nympl	2	80	60	66.67
	adult	3	60	70	60
B. bassiar	2nd instar nymj	4	100	77.78	88.89
	5th instar nympl	5	80	66.67	80
	adult	6	60	66.67	70

SPSS17.0

描述

VAR00002

		N	均值	标准差	标准误	均值的 95% 置信区间 下限	均值的 95% 置信区间 上限	极小值	极大值	分量间方差
1.00		3	78.1481	11.67108	6.73830	49.1556	107.1407	66.67	90.00	
2.00		3	68.8889	10.18350	5.87945	43.5917	94.1861	60.00	80.00	
3.00		3	63.3333	5.77350	3.33333	48.9912	77.6755	60.00	70.00	
4.00		3	88.8889	11.11111	6.41500	61.2874	116.4904	77.78	100.00	
5.00		3	75.5556	7.69800	4.44444	56.4327	94.6785	66.67	80.00	
6.00		3	65.5556	5.09175	2.93972	52.9069	78.2042	60.00	70.00	
总数		18	73.3951	11.66883	2.75037	67.5923	79.1978	60.00	100.00	
模型	固定效应			8.96189	2.11234	68.7927	77.9975			
	随机效应				3.87436	63.4357	83.3544			63.29218

ANOVA

VAR00002

			平方和	df	均方	F	显著性
组间	（组合）		1350.960	5	270.192	3.364	.039
	线性项	对比	12.986	1	12.986	.162	.695
		偏差	1337.974	4	334.493	4.165	.024
组内			963.786	12	80.316		
总数			2314.746	17			

多重比较

因变量:VAR00002

	(I) VAR00001	(J) VAR00001	均值差 (I-J)	标准误	显著性	95% 置信区间 下限	95% 置信区间 上限
LSD	1.00	2.00	9.25926	7.31735	.230	-6.6839	25.2024
		3.00	14.81481	7.31735	.066	-1.1283	30.7580
		4.00	-10.74074	7.31735	.168	-26.6839	5.2024
		5.00	2.59259	7.31735	.729	-13.3506	18.5357
		6.00	12.59259	7.31735	.111	-3.3506	28.5357
	2.00	1.00	-9.25926	7.31735	.230	-25.2024	6.6839
		3.00	5.55556	7.31735	.462	-10.3876	21.4987

		[illegible]	[illegible]	[illegible]	[illegible]	[illegible]	[illegible]
		4.00	-20.00000*	7.31735	.018	-35.9431	-4.0569
		5.00	-6.66667	7.31735	.380	-22.6098	9.2765
		6.00	3.33333	7.31735	.657	-12.6098	19.2765
	3.00	1.00	-14.81481	7.31735	.066	-30.7580	1.1283
		2.00	-5.55556	7.31735	.462	-21.4987	10.3876
		4.00	-25.55556*	7.31735	.004	-41.4987	-9.6124
		5.00	-12.22222	7.31735	.121	-28.1654	3.7209
		6.00	-2.22222	7.31735	.767	-18.1654	13.7209
	4.00	1.00	10.74074	7.31735	.168	-5.2024	26.6839
		2.00	20.00000*	7.31735	.018	4.0569	35.9431
		3.00	25.55556*	7.31735	.004	9.6124	41.4987
		5.00	13.33333	7.31735	.093	-2.6098	29.2765
		6.00	23.33333*	7.31735	.008	7.3902	39.2765
	5.00	1.00	-2.59259	7.31735	.729	-18.5357	13.3506
		2.00	6.66667	7.31735	.380	-9.2765	22.6098
		3.00	12.22222	7.31735	.121	-3.7209	28.1654
		4.00	-13.33333	7.31735	.093	-29.2765	2.6098
		6.00	10.00000	7.31735	.197	-5.9431	25.9431
	6.00	1.00	-12.59259	7.31735	.111	-28.5357	3.3506
		2.00	-3.33333	7.31735	.657	-19.2765	12.6098
		3.00	2.22222	7.31735	.767	-13.7209	18.1654
		4.00	-23.33333*	7.31735	.008	-39.2765	-7.3902
		5.00	-10.00000	7.31735	.197	-25.9431	5.9431

DPS

计算结果	当前日期 2017-8-23 PM 12:06:14						
处理	样本数	均值	标准差	标准误	95%置信区间		
1	3	78.15	11.6694	6.7373	49.1616	107.1384	
2	3	68.89	10.1831	5.8792	43.5937	94.1863	
3	3	63.3333	5.7735	3.3333	48.9912	77.6755	
4	3	88.89	11.11	6.4144	61.2912	116.4888	
5	3	75.5567	7.6961	4.4433	56.4385	94.6748	
6	3	65.5567	5.0921	2.9399	52.9072	78.2062	
	方差分析表						
变异来源	平方和	自由度	均 方	F 值	p值		
处理间	1351.0484	5	270.2097	3.365	0.0395		
处理内	963.5919	12	80.2993				
总变异	2314.6403	17					

LSD法多重比较(下三角为均值差,上三角为显著水平)							
No.	均值	4	1	5	2	6	3
4	88.89		0.1679	0.0934	0.0181	0.0078	0.0044
1	78.15	10.74		0.7292	0.2297	0.1109	0.0657
5	75.5567	13.3333	2.5933		0.3801	0.1968	0.1207
2	68.89	20	9.26	6.6667		0.6568	0.4622
6	65.5567	23.3333	12.5933	10	3.3333		0.7664
3	63.3333	25.5567	14.8167	12.2233	5.5567	2.2233	
字母标记表示结果							
处理	均值	5%显著水平		1%极显著水平			
4	88.89	a		A			
1	78.15	ab		AB			
5	75.5567	ab		AB			
2	68.89	b		AB			
6	65.5567	b		B			
3	63.3333	b		B			

average mortality

			mortality(from raw data)		
			repetition1	2	3
P. lilacinu	2nd instar nymph	1	90	80	70
	5th instar nymph	2	80	60	70
	adult	3	60	70	60
B. bassian	2nd instar nymph	4	100	80	90
	5th instar nymph	5	80	70	80
	adult	6	60	70	70

SPSS17.0

VAR00002

		N	均值	标准差	标准误	均值的 95% 置信区间 下限	上限	极小值	极大值	分量间方差
1.00		3	80.0000	10.00000	5.77350	55.1586	104.8414	70.00	90.00	
2.00		3	70.0000	10.00000	5.77350	45.1586	94.8414	60.00	80.00	
3.00		3	63.3333	5.77350	3.33333	48.9912	77.6755	60.00	70.00	
4.00		3	90.0000	10.00000	5.77350	65.1586	114.8414	80.00	100.00	
5.00		3	76.6667	5.77350	3.33333	62.3245	91.0088	70.00	80.00	
6.00		3	66.6667	5.77350	3.33333	52.3245	81.0088	60.00	70.00	
总数		18	74.4444	11.49026	2.70828	68.7305	80.1584	60.00	100.00	
模型	固定效应			8.16497	1.92450	70.2513	78.6376			
	随机效应				4.00617	64.1463	84.7426			74.07407

ANOVA

VAR00002

			平方和	df	均方	F	显著性
组间	(组合)		1444.444	5	288.889	4.333	.017
	线性项	对比	17.143	1	17.143	.257	.621
		偏差	1427.302	4	356.825	5.352	.010
组内			800.000	12	66.667		
总数			2244.444	17			

多重比较

因变量:VAR00002

	(I) VAR00001	(J) VAR00001	均值差 (I-J)	标准误	显著性	95% 置信区间 下限	上限
LSD	1.00	2.00	10.00000	6.66667	.159	-4.5254	24.5254
		3.00	16.66667*	6.66667	.028	2.1412	31.1921
		4.00	-10.00000	6.66667	.159	-24.5254	4.5254
		5.00	3.33333	6.66667	.626	-11.1921	17.8588
		6.00	13.33333	6.66667	.069	-1.1921	27.8588
	2.00	1.00	-10.00000	6.66667	.159	-24.5254	4.5254
		3.00	6.66667	6.66667	.337	-7.8588	21.1921
		[illegible]	[illegible]*	[illegible]	[illegible]	[illegible]	[illegible]

		4.00	-20.00000*	6.66667	.011	-34.5254	-5.4746
		5.00	-6.66667	6.66667	.337	-21.1921	7.8588
		6.00	3.33333	6.66667	.626	-11.1921	17.8588
	3.00	1.00	-16.66667*	6.66667	.028	-31.1921	-2.1412
		2.00	-6.66667	6.66667	.337	-21.1921	7.8588
		4.00	-26.66667*	6.66667	.002	-41.1921	-12.1412
		5.00	-13.33333	6.66667	.069	-27.8588	1.1921
		6.00	-3.33333	6.66667	.626	-17.8588	11.1921
	4.00	1.00	10.00000	6.66667	.159	-4.5254	24.5254
		2.00	20.00000*	6.66667	.011	5.4746	34.5254
		3.00	26.66667*	6.66667	.002	12.1412	41.1921
		5.00	13.33333	6.66667	.069	-1.1921	27.8588
		6.00	23.33333*	6.66667	.004	8.8079	37.8588
	5.00	1.00	-3.33333	6.66667	.626	-17.8588	11.1921
		2.00	6.66667	6.66667	.337	-7.8588	21.1921
		3.00	13.33333	6.66667	.069	-1.1921	27.8588
		4.00	-13.33333	6.66667	.069	-27.8588	1.1921
		6.00	10.00000	6.66667	.159	-4.5254	24.5254
	6.00	1.00	-13.33333	6.66667	.069	-27.8588	1.1921
		2.00	-3.33333	6.66667	.626	-17.8588	11.1921
		3.00	3.33333	6.66667	.626	-11.1921	17.8588
		4.00	-23.33333*	6.66667	.004	-37.8588	-8.8079
		5.00	-10.00000	6.66667	.159	-24.5254	4.5254
Tamhane	1.00	2.00	10.00000	8.16497	.994	-40.7354	60.7354
		3.00	16.66667	6.66667	.725	-35.6241	68.9574

DPS

计算结果	当前日期 2017-8-23 PM 12:17:23							
处理	样本数	均值	标准差	标准误	95%置信区间			
1	3	80	10	5.7735	55.1586	104.8414		
2	3	70	10	5.7735	45.1586	94.8414		
3	3	63.3333	5.7735	3.3333	48.9912	77.6755		
4	3	90	10	5.7735	65.1586	114.8414		
5	3	76.6667	5.7735	3.3333	62.3245	91.0088		
6	3	66.6667	5.7735	3.3333	52.3245	81.0088		
	方差分析表							
变异来源	平方和	自由度	均 方	F 值	p值			
处理间	1444.444	5	288.8889	4.333	0.0174			
处理内	800	12	66.6667					
总变异	2244.444	17						

LSD法多重比较(下三角为均值差,上三角为显著水平)							
No.	均值	4	1	5	2	6	3
4	90		0.1595	0.0687	0.0111	0.0044	0.0018
1	80	10		0.6261	0.1595	0.0687	0.0279
5	76.6667	13.3333	3.3333		0.337	0.1595	0.0687
2	70	20	10	6.6667		0.6261	0.337
6	66.6667	23.3333	13.3333	10	3.3333		0.6261
3	63.3333	26.6667	16.6667	13.3333	6.6667	3.3333	
字母标记表示结果							
处理	均值	5%显著水平		1%极显著水平			
4	90	a		A			
1	80	ab		AB			
5	76.6667	abc		AB			
2	70	bc		AB			
6	66.6667	bc		B			
3	63.3333	c		B			
