# Supplementary material for: The isolation and identification of pathogenic fungi from Tessaratoma papillosa Drury (Hemiptera: Tessaratomidae)
Source: PeerJ. 2017 Oct 6;5:e3888. doi: 10.7717/peerj.3888 (PMC5633030; doi:10.7717/peerj.3888)
Supplement: Supplemental Information 4 [file peerj-05-3888-s004.docx]

| TA-01 | Tatacaactcccaaacccctgtgaaccttacctcagttgcctcggcgggaacgccccggccgcctgcccccgcgccggcgccggacccaggcgcccgccgcagggaccccaaactctcttgcattacgcccagcgggcggaatttcttctctgagttgcacaagcaaaaacaaatgaatcaaaactttcaacaacggatctcttggttctggcatcgatgaagaacgcagcgaaatgcgataagtaatgtgaattgcagaattcagtgaatcatcgaatctttgaacgcacattgcgcccgccagcattctggcgggcatgcctgttcgagcgtcatttcaaccctcgagcccccccgggggcctcggtgttgggggacggcacaccagccgcccccgaaatgcagtggcgaccccgccgcagcctcccctgcgtagtagcacacacctcgcaccggagcgcggaggcggtcacgccgtaaaacgcccaactttcttagagttgacctcggatcaggtaggaatacccgctgaacttaagcatatcaaaagccggagaa |
| --- | --- |
| TA-02 | GCCTCAACCCTTCTGTGAACCTACCTATCGTTGCTTCGGCGGACTCGCCCCAGCCCGGACGCGGACTGGACCAGTGGCCCGCCGGGGACCTCAAACTCTTGTATTCCAGCATCTTCTGAATACGCCGCAAGGCAAAACAAATGAATCAAAACTTTCAACAACGGATCTCTTGGCTCTGGCATCGATGAAGAACGCAGCGAAGCGCGATAAGTAATGTGAATTGCAGAATCCAGTGAATCATCGAATCTTTGAACGCACATTGCGCCCGCCAGCATTCTGGCGGGCATGCCTGTTCGAGCGTCATTTCAACCCTCGACCTCCCCTTGGGGAGGTCGGCGTTGGGGACCGGCAGCACACCGCCGGCCCTGAAATGGAGTGGCGGCCCGTCCGCGGCGACCTCTGCGCAGTAATACAGCTCGCACCGGGACCCCGACGCGGCCACGCCGGAAAA |
